# A Review of Arguments for the Existence of Latent Infections of *Bacillus anthracis*, and Research Needed to Understand Their Role in the Outbreaks of Anthrax

**DOI:** 10.3390/microorganisms8060800

**Published:** 2020-05-26

**Authors:** Robert S. Gainer, Gilles Vergnaud, Martin E. Hugh-Jones

**Affiliations:** 1 Herb’s Institute, Box 1058, Hanna, AB T0J 1P0, Canada; 2 Institute for Integrative Biology of the Cell (I2BC), Université Paris-Saclay, 91198 Gif-Sur-Yvette, France; gilles.vergnaud@universite-paris-saclay.fr; 3 Department of Environmental Sciences, College of the Coast and Environment, Louisiana State University, Baton Rouge, LA 70803-5705, USA; mhughj1@lsu.edu

**Keywords:** anthrax, latent infections, modified host resistance, tabanids

## Abstract

Hugh-Jones and Blackburn and Turnbull’s collective World Health Organization (WHO) report did literature reviews of the theories and the bases for causes of anthrax outbreaks. Both comment on an often-mentioned suspicion that, even though unproven, latent infections are likely involved. Hugh-Jones suggested Gainer do an updated review of our present-day knowledge of latent infections, which was the basis for Gainer’s talk at the Biology of Anthrax Conference in Bari, Italy 2019. At the Conference Gainer met Vergnaud who presented anthrax genome studies that implied that the disease might have spread throughout Asia and from Europe to North America in a short time span of three or four centuries. Vergnaud wondered if latent infections might have played a role in the process. Several other presenters at the Conference also mentioned results that might suggest the existence of latent infections. Vergnaud subsequently looked into some of the old French literature about related observations, results, and discussions of early Pasteur vaccine usage (late 1800′s) and found mentions of suspected latent infections. The first part of the paper is a focused summary and interpretation of Hugh-Jones and Blackburn’s and Turnbull’s reviews specifically looking for suggestions of latent infections, a few additional studies with slightly different approaches, and several mentions made of presentations and posters at the Conference in Italy. In general, many different investigators in different areas and aspects of the anthrax study at the Conference found reasons to suspect the existence of latent infections. The authors conclude that the affected species most studied, including *Homo sapiens*, provide circumstantial evidence of latent infections and modified host resistance. The last part of the review explores the research needed to prove or disprove the existence of latent infections.

## 1. Introduction

At the International Conference on the Biology of Anthrax (Bari, Italy, 2019), Martin Hugh-Jones concluded that the more than 100 years of studies on *Bacillus anthracis* and of the anthrax disease still leave many unanswered questions about what caused several of the outbreaks. Hugh-Jones and Blackburn’s (2009) review ”Ecology of Anthrax” [1] has a conclusion and discussion with four points that need explanation (summarized): 1. Spore non-survival in low pH soils. 2. The dosage of spores actually available in outbreak areas. 3. Although tabanids are prominent in many known outbreak areas involving cattle, no experimental transmission of the disease by tabanids has been achieved so far. 4. Latent infections have not been proven. Turnbull [2] also reviews the etiology of the disease and states that there are still many anomalies and unknowns about our understanding of the disease. The much more extensive and referenced discussions in these two documents are paraphrased in the present review. In particular, Turnbull’s collective work [2] (pages 26–27), more than 10 years ago, linked a significant part of these anomalies to our modest understanding of a number of features regarding *B. anthracis* interaction with its hosts including prolonged incubation, carrier state, unapparent, chronic and latent infections. In the rest of this review, we will use the term “latent infection” to designate all of these categories (latent infections in the broad sense, *sensu lato; sensu stricto* would refer to the disease contained in an inactive state by the immune system).

## 2. Sources of Infection: Spores

Like Hugh-Jones and Blackburn [1], Turnbull [2] also considers that the numbers of spores experimentally required to cause infections are rarely reflected in the field, especially considering the large doses needed to cause the peracute form [3,4,5]. Talks at the Conference by Hassim, Bennett, Jordan, and Baillie also reflected this. One reason may be our misunderstanding of spores. Turnbull [2] (page 11) considers *B. anthracis* an obligate pathogen that must sporulate or die outside its host. Sporulation is a much longer and more demanding process than germination. Under ideal circumstances, sporulation takes a minimum of twelve hours to complete and germination takes 2–10 min. Spore maintenance (non-germination) requires dryness and an alkaline pH even though Turnbull [2] (page 14) points out that the low temperature of Gruinard Island likely accounted for the preservation of spores in its acid soils.

Once spores have formed, however, most soils will have periods of dampness (and/or other conditions), which will promote their germination. The resulting vegetative state cannot stand adverse pH, sunlight, other soil chemicals, etc. and is easily outcompeted and eliminated by better adapted soil microorganisms [6]. Some studies have suggested that water erosion can concentrate spores. However, water is conducive to spore germination and its exosporium actually seems to prevent dispersion [2] (page 60). Others point out that if there is a concentration of spores, there will be a gradient of dosages whereas outbreaks are mainly characterized by peracute infections.

Turnbull [2] (page 18) highlights the anomaly of many investigations on outbreaks of anthrax in cattle, which are the most prone and commonly affected domestic species but also experimentally highly resistant. Hundreds of spores are required orally to cause a minimum infection with certainty. Yet, in the field, during outbreaks involving cattle, such amounts are not detected. Tabanids, that experimentally can cause only inconsequential localized infections, are usually rampant. Although the sudden death, peracute form of the disease, requiring high doses of spores orally, is most recognized, serological studies have shown that a significant portion of the rest of the population had developed antibodies to anthrax.

## 3. The Environment: Modified Host Resistance

Conditions that Gainer [4,5] associated with several anthrax outbreaks in East African Game Reserves and Parks during the early 1970s were often harsh. With grazing animals that seasonally congregate, they usually included heat, drought, overgrazing, breeding, overpopulation, and extreme insect harassment (especially from tabanids). In Wood Buffalo National Park and environs, climate and food supply are at their best during the summer but the insect harassment is at its most extreme.

Turnbull [2] (pages 15, 40) suggested that an overdependence on the LD50 dose-dependency experiments may have influenced investigators into looking for large numbers of spores in the soil rather than for the effects of the environment on the host. He points out that the “stress theory” and immunocompetence of the host is not new, and poor environmental conditions and tabanids were mentioned as influencing factors by Hutyra et al. (1946) [7] as well as other veterinarians starting back in Pasteur’s time [8,9,10]. Stein [11] quoted several American veterinary practitioners involved in anthrax control measures during the 1950s as saying they thought that contributing factors to the outbreaks were tabanids and other stressful environmental conditions for cattle. Provost et al. (1974) [12] conducted immunological tests and cultures in West Africa on cattle from anthrax areas showing that a high proportion of the population had survived exposure to the disease. They also remarked on the stressful environmental conditions during outbreaks. Hugh-Jones and Blackburn [1] make several references to climatic and environmental conditions during outbreaks, especially drought, starvation, and intense tabanid activity, and the stress (and modification of host resistance) this must impose on the indigenous animals.

For ethical reasons, none of the experiments for the LD50 infective dosages would have been done on animals that were stressed. As stress goes down, immune system function goes up. This might explain why in the experiments such large oral dosages (parenteral infections were inconsequential) were needed while, in the field where there are stressful conditions and only a few spores, peracute infections occur and tabanids greatly enlarge the outbreak.

Kolonin [13,14], similarly to Hugh-Jones and Blackburn [1], combined geography and veterinary control measures and noticed a pattern to anthrax distribution in the former Soviet Union. Reports of the disease did not reoccur as much, and, in fact, often stopped after vaccination programs in the north, but they would reoccur in the south. He proposed that, in contrast to southern alkaline steppe soils, the acid taiga soils in the north did not allow the persistence of spores. He also noted that the north had a much more extreme insect harassment, especially the June 15-July 15 tabanid season, compared to the south, with a clear effect on the well-being and immune status of people and animals. He postulated that the source of infection in the north was from spores in the small areas of alkaline soil where domestic animals were kept. The peracute infections that developed in a few animals would be spread by tabanids and the stress due to the insects would reduce the animals’ resistance to anthrax infection. Reindeers have suffered massively from anthrax outbreaks. Soviets vaccinated domestic animals in Yamal from 1925–1945 and the disease disappeared for 75 years until a minor outbreak in 2016 [15].

Gainer’s two trips to Yamal in 2016 and over ten years of livestock raising and veterinary practice in Wood Buffalo National Park and environs finds the two regions to be similar. Both are taiga and have enormous rivers and alkaline floodplains. There are millions of reindeer in Yamal compared to the few thousand bison in Wood Buffalo environs. It cannot be overemphasized how distressing the insect harassment and not being able to escape it can be in both environments [16].

Turnbull [2] (pages 16, 23, 24) reviewed the suspected importance of tabanids in other anthrax outbreaks, including early references such as Budd (1893) [17], Minett, and Dhanda (1941) [18]. Davies involved tabanids in the huge 1978–1979 Zimbabwe outbreak [19]. Demonstrating the role of tabanids in the field is not practical, but Turell et al. [20] demonstrated mechanical transmission and Blackburn et al. [21] collected *B. anthracis* from biting flies during an anthrax season. Blackburn et al. [22] presented empirical and genetic evidence of biting fly transmission during an anthrax outbreak, and Mullins et al. [23] did a spatial and temporal analysis of biting flies during the anthrax season in West Texas, which implicates their involvement in the disease (answering Hugh-Jones and Blackburn’s third question [1]). Fasanella et al. [24] suspected fly transmission from sheep in a human cutaneous anthrax case. Transmission of the infection is considered unproven because experiments in cattle indicated that parenteral infections can only cause a localized infection of no consequence. During the 2001 anthrax epidemic in the West Texas deer ranches, some ranches lost up to 80% of their white-tailed deer, which is much more than nearby livestock losses. White tails, like bison and reindeer, may be more prone to anthrax because they are more targeted by tabanids or have a different relationship with tabanids, or have a more susceptible genetic background.

## 4. Latent Infections

Ferguson (1981) [25] described chronic infections of retropharyngeal and gastric lymph nodes in pigs from which *B. anthracis* could be cultured by meat inspectors in the American Midwest during the 1950s. Ante mortem, the pigs were healthy, but anthrax outbreaks had been widespread in cattle several months earlier and the pigs were fed contaminated bone meal from the carcasses. Provost et al. [9] cultured presumed *B. anthracis* from the retropharyngeal lymph nodes of otherwise healthy cattle from anthrax areas in West Africa. Turnbull [2] (pages 26–27) reviews prolonged incubation, carriage, and carrier states as well as unapparent, chronic, and latent infections. All of these conditions have been recognized and described in several species for more than 100 years, including humans. Provost and Trouette (1957) referred to the book by Nocard and Leclainche [8], and observations by Besredka who indicated that *B. anthracis* might remain cryptic in the host and be activated if the host is weakened. Bigoteau [10], using the newly developed Pasteur vaccines, reported in 1893 that he was much more likely to see anthrax accidents after vaccination in herds in which anthrax had occurred recently when compared to herds with no known history of anthrax. The author concluded that a subclinical infection was activated by the vaccine, eliminating a fraction of the animals. In two instances reported by Bigoteau, about ten among 100 sheep of a cohort with a preexisting history of anthrax died of anthrax after the second vaccine injection, whereas no incidents were observed in the other cohorts vaccinated at the same time [10]. According to Bigoteau, the adverse effect of the Pasteur vaccine was an indicator of the presence of latent infections (a description of anthrax latent infections 130 years ago). The role Pasteur vaccine (including two strains, one of which contains both virulence plasmids) might play in revealing the existence of latent infections is currently unexplained. Kolonin [13,14] thought the Russian vaccine and elimination of carcasses must have eliminated latent infections. Turnbull [2] (page 13) thought vaccination and elimination of carcasses also eliminated latent infections in most areas. The apparent efficiency of the Office International des Epizooties (OIE) 20 days quarantine may be a strong argument against the existence of latent infections. However, animals selected for exports are in the best condition before, during, and after the quarantine, are usually vaccinated regularly, and may be given antibiotics prophylactically. All these measures would have reduced or eliminated latent infections.

Vergnaud’s presentation was on the spread of *B. anthracis* lineages throughout Eurasia [26]. The implication was that military operations have played a major role in the spread of *B. anthracis* in agreement with the inclusion of anthrax among war-associated diseases [27]. Along this line, he proposed that the ancestor of the currently well recognized lineage in North America was introduced from France to Canada by the Carignan-Salieres regiment in 1665, which is a major and unique French military operation during the 17th century [28,29,30]. This operation also coincides with the introduction of the first batch of 12 horses among 80, which will eventually produce the “Canadian” breed [31]. Latent infections could provide a convenient way to explain the introduction of the disease to North America. Hudson Bay employees recorded die offs in the Wood Buffalo National Park area in the early 1800s and Palliser [32] (page 194) records native reports of bison and ungulate die offs in the 1850s.

Latent infections do not have easily recognized symptoms that several presenters at the Conference commented on. In Jordan, Aqel reported the livestock moves freely across their borders with countries that do not recognize or control the disease. Doganay and Baillie made similar remarks regarding Turkey. There were also lineages showing up where they were not before. Hoffmaster talked about this in California and a Texas hunt ranch, Muller in Australia, and Lekota and Hassim in Southern Africa. Spores are easily transported by agricultural products in general, but in low numbers and poor places where the chance of causing an infection before their elimination is low. Latent infections, however, would be in place and ready for the immune system to let them become peracute [10].

Hugh-Jones remarked that latent infections would be an evolutionary advantage to a pathogen that developed from a soil bacterium. Spore storage and transmission would have been secondary. The relationship that anthrax has with some host species must inevitably involve the host’s immune system, latent infections, stressful environmental conditions, and mutual benefit. For instance, Gainer’s [33,34] study of anthrax in wildebeest in the Selous Game Reserve in Tanzania in the early 1970s (at the time isolated from livestock and domestication) might illustrate such a relationship. The region’s carnivores all moved to the wildebeest calving area where the wildebeest had concentrated to take advantage of the lush green short grass. During this very hot and humid period, tabanids were oppressive and anthrax was rampant. Rather than predate the young, the carnivores would scavenge the anthrax carcasses. Recruitment more than offset the anthrax mortality. The overall wildebeest population was thriving, actually exceeding its resource capacity with significant areas of over grazing. However, overall, the grazing resulted in an expanded area of shortgrass at the expense of the surrounding shrub land. The more wildebeests, the shorter the grass area, the more *B. anthracis*, the more carnivores present, the more tabanids, etc., which is a simple density dependent feedback loop based on the immune system. Other wildebeest populations with anthrax, but with human disturbance, did not seem to have a mutualistic relationship [35].

In humans, cutaneous anthrax represents 90%–95% of cases, the enteric form represents 5%–9%, and the inhalation form represents less than 1% [2] (p42:4.3.2). Cutaneous anthrax, although potentially fatal, is more often self-limiting when left untreated (p43:4.4.1). During pre-antibiotic days, it is estimated that 10%–40% of cases were fatal. Mild cases in much of the third world today still go untreated (p43:4.4.1). Serological and epidemiological evidence suggests that undiagnosed low grade or subclinical anthrax infection (of unknown form) is not rare and spontaneous recovery is common (p43.4.4.1).

Enteric anthrax, usually from the consumption of poorly cooked infected meat, is more likely to be lethal, but this is not easily evaluated. Most cases of gastroenteritis will attract little attention, are treated non-specifically, and recover [2] (p46–47). Infrequently, anthrax is diagnosed and treated with an appropriate antibiotic (p38, p43). More frequently, it is a postmortem diagnosis. If antibiotics were not used and a relapse with a subsequent diagnosis of anthrax is made, some physicians invoke a latent infection. Records of second infections are very rare [2] (p51:4.4.8) with no possibility to distinguish reactivation of a latent infection from second independent exposures. The possibilities offered by whole genome sequencing technologies might allow more precise investigation of such cases, at least if systematic sequencing becomes commonplace.

Nakanwagi et al. [36] describe enteric anthrax in about 60 people presented with and treated for non-specific gastroenteritis in 2017 in Uganda that, subsequently, turned out to be anthrax. Despite the fact that the One Health multisectoral committee ranked anthrax as its highest priority zoonotic disease, this was the only documentation of anthrax in humans in this area. This outbreak illustrated that meat from anthrax carcasses is still likely to end up in the local markets if not in the owner’s own kitchen, as already described as common practice in ancient literature [37], but also by Aqel, Doganay, and Baillie at the Conference, and the hides go to the tanneries. Bennett talked about African drum groups using hides from anthrax animals having a variable effect on users.

Acute inhalation anthrax is usually a post mortem diagnosis. If suspected, an early response to treatment is possible. Historically, it has long been suspected with some supportive evidence that undiagnosed low-grade inhalation infections must occur in at-risk occupations and provide workers immunity from higher dosages [2] (p49:4.4.4.1). Serological surveys suggest it may be more common [2] (p.45:4.4.2.2.), and it has been stated that people can recover without antibiotics, from the acute stage, even though this is likely the exception [2] (p43:4.4.1). Baillie also talked about studies of wool sorters’ exposure to inhalation anthrax showing how resistant most people were, depending on their health. Bower and Dupke had posters about human serological surveillance in Côte d’Ivoire. Laws talked about using almost 30 people who had a history of cutaneous anthrax in his study of human response to vaccination with some as long as eight years after the infection. However, regardless of time after infections, they all responded similarly. Hugh-Jones and Blackburn (2009) [1] mentioned that humans have great variation in resistance to the disease. A homeless drug addict succumbed to anthrax several weeks after the Sverdlovsk accident [38,39].

The most significant point regarding latent infections comes from more than a century of physicians diagnosing, discussing, advising, and describing anthrax infections with their patients and colleagues. Anthrax in humans is an occupational hazard. Contraction of the disease depends significantly upon the health of the patient’s immune system, which is very dependent upon the patient’s lifestyle and personal choices. Physicians that have seen or suspected a patient that recovered from the disease without the use of antibiotics recognize that the immune system must have improved and reduced the infection into a form of latency that gradually disappeared with time. This is likely the most compelling evidence of latent infections.

Every situation in which an anthrax outbreak has been studied is somewhat different from the others. The reason for this may be that the disease strongly depends upon a combination of external and internal environmental factors. Every outbreak uses these factors in different ways. The multisectoral One Health approach by human public health, and veterinary organizations seemed to be emphasized in Turnbull’s document and at the conference. This discussion felt that the single factor model of LD50 and infectivity only explains experimental results. For all the different forms of anthrax infections, a combination of low spore numbers or parenteral infections, stressful host conditions, and modified host resistance is needed, which usually happens with other diseases. More than ten years ago, Turnbull [2] (pages 14–15) emphasized that the external environment had been much better studied than the internal environment.

At this point in our knowledge, or ignorance, of *B. anthracis* latency, it would be safe to assume it is not uncommon but that recognition depends on those in unusual circumstances of isolation. For example:

1.  Isolation in Space:
  a: Seroconverting wood bison in the Mackenzie Bison Sanctuary that display seropositivity in the spring blood testing and in the absence of any known prior deaths in the previous 12–24 months in the area.
  b: Livestock that had been transported long distances.
2.  Isolation in Time:

A member of a herd that had been subjected to an anthrax outbreak, grazing in a different meadow, succumbs the following year in the middle to late summer during the hot weather and loss of innate resistance.

3. Vaccination accident in endemic countries:

The early investigations by Bigoteau [10] suggesting that, in an endemic country, some herds might be hosting *B. anthracis* and that this will be manifested by an abnormally high rate of vaccination accidents not necessarily fatal would need to be confirmed. In particular, one might expect that if the vaccine is activating a latent infection, strains that would be recovered will be distinct from the vaccine strain. Whole genome sequencing would allow to make the difference.

While there is circumstantial epidemiological evidence that latent infections might occur, their frequency is unclear and there is a need for agreed criteria for confirmation and definition, which will provide enumeration. If one were looking for latent infections in white tail deer during the December hunting season following a summer with a number of deer deaths from anthrax, the presence of clinical cases in December would allow a presumption of latent infection. When infected pig lymph nodes were noted at slaughter in an abattoir [11], which was 6–9 months after a widespread porcine epidemic due to contaminated feed, the meat inspectors noted that the lymph nodes were inflamed and wet. Was this characteristic of only this event or is lymph node inflammation common with latent infections? If there are spores embedded, it might indicate feed exposure. If vegetative cells, it might indicate a subclinical bacteremia. A decision will have to be made as to whether to hold back part of each lymph node unfixed, whatever their culture status, so that they can later be checked by PCR for the presence of missed organisms.

Clearly, research is needed to test a latent infection hypothesis. First, it should aim to define the pathology of a *B. anthracis* latent infection. The target order may be as follows: (1) estimate the prevalence and nature of latent infections in west Texas white tail deer following an anthrax outbreak by utilizing the ranch carcass dressing station(s) to sample deer shot in the subsequent hunting season whatever their apparent status, and (2) similarly investigate herd members from cattle ranches with anthrax cases when some are slaughtered in the following December to March.
